# MoS_2_-Nanoflower and Nanodiamond Co-Engineered Surface Plasmon Resonance for Biosensing

**DOI:** 10.3390/bios13050506

**Published:** 2023-04-28

**Authors:** Yaofei Chen, Xin Xiong, Yu Chen, Lei Chen, Guishi Liu, Wei Xiao, Jifu Shi, Zhe Chen, Yunhan Luo

**Affiliations:** 1 Guangdong Provincial Key Laboratory of Optical Fiber Sensing and Communications, Jinan University, Guangzhou 510632, China; chenyaofei@jnu.edu.cn (Y.C.); xiongxin@stu2020.jnu.edu.cn (X.X.);; 2 Department of Optoelectronic Engineering, Jinan University, Guangzhou 510632, China; 3 Department of Laboratory Medicine, Guangdong Second Provincial General Hospital, Guangzhou 510317, China; 4 Siyuan Laboratory, Department of Physics, Jinan University, Guangzhou 510632, China

**Keywords:** SPR-based biosensor, MoS_2_ nanoflowers, Nanodiamonds, surface engineering, sensitivity enhancement

## Abstract

Surface plasmon resonance (SPR) based sensors play an important role in the biological and medical fields, and improving the sensitivity is a goal that has always been pursued. In this paper, a sensitivity enhancement scheme jointly employing MoS_2_ nanoflower (MNF) and nanodiamond (ND) to co-engineer the plasmonic surface was proposed and demonstrated. The scheme could be easily implemented via physically depositing MNF and ND overlayers on the gold surface of an SPR chip, and the overlayer could be flexibly adjusted by controlling the deposition times, thus approaching the optimal performance. The bulk RI sensitivity was enhanced from 9682 to 12,219 nm/RIU under the optimal condition that successively deposited MNF and ND 1 and 2 times. The proposed scheme was proved in an IgG immunoassay, where the sensitivity was twice enhanced compared to the traditional bare gold surface. Characterization and simulation results revealed that the improvement arose from the enhanced sensing field and increased antibody loading via the deposited MNF and ND overlayer. At the same time, the versatile surface property of NDs allowed a specifically-functionalized sensor using the standard method compatible with a gold surface. Besides, the application for pseudorabies virus detection in serum solution was also demonstrated.

## 1. Introduction

Biological analytes detection is significant research because it plays a vital indicator for biomedical evaluation. With the development of modern medical testing technology, optical and electronic sensors to detect extremely dilute concentrations and small molecular weights of analytes have raised great interest in recent years [1]. As one kind of optical sensor, the sensors based on surface plasmon resonance (SPR) have shown great application potential in the detection of biochemical molecules due to their advantages of label-free, real-time, online monitoring, etc. [2]. SPR is a physical phenomenon that occurs at the interface of a dielectric and metal; the surface Raman scattering (SERS) is enhanced by the metal layer on the surface [3], and it is very sensitive to medium refractive index (RI) changes of sensing layer [4,5]. However, improving the sensitivity of SPR sensors is a goal that has always been pursued regarding detecting biomolecules with small weight or ultra-low concentration [6].

It has been proved that well-engineering the plasmonic surface by nanostructure or nanomaterial can effectively improve sensitivity [7,8]. One of the commonly-adopted schemes is to replace the conventional single metal film with a well-designed metal nanoarray structure, such as nanostrips [9] and nanorods [10] on the metal layer. In this way, the sensor’s sensitivity can be improved to a high level of 10,000–30,000 nm/RIU [11,12]. Moreover, the metal-insulator-metal (MIM) waveguide can form a double-cavity structure, providing the potential for temperature compensation by monitoring the two resonance wavelengths [13]. In these cases, the sensing field on the sensor surface was enhanced by the polariton coupling from the additional nanostructure, significantly increasing the penetration depth and sensitivity. However, the fabrication of these nanostructures needs complicated processes, such as electron-beam lithography. Using a multilayer thin-film structure, such as a hyperbolic metamaterial composed of alternant metal/dielectric films [14,15,16], can alleviate the complicated process. Our recent work demonstrated that tuning the angle of incidence can achieve large penetration depths to 1800 nm, yielding a theoretical sensitivity of ~10^5^ nm/RIU [17]. An alternative and easy implementation scheme is attaching nanomaterials to the surface of a metal plasmonic layer by chemical modification or physical deposition [18]. Different forms of nanomaterials, including nanoparticles (such as Au [19] and TiO_2_ [20]), nanotubes, such as halloysite [21], two-dimensional materials (such as graphene oxide [22,23], MoSe_2_ [24], GeSe [25], Mxene [26], antimonene [27]), and so on have been demonstrated to improve SPR sensitivity. MoS_2_ is a cheap and abundant mineral, and its nanocomposites with Ni and gold are used in electronic, magnetic, and catalytic applications [28]. Our recent works [29,30] showed that depositing MoS_2_ nanoflower (MNF) on a metal film surface not only improved the bulk RI sensitivity via engineering the plasmonic coupling condition but also provided more loading sites for biological probes, which is beneficial to high-sensitivity biological sensing.

In this paper, we proposed a scheme using MNF and nanodiamond (ND) to co-engineer the plasmonic surface for sensitivity-enhanced biosensing. In this scheme, the advantages of MNF and ND were combinedly exploited. MNFs can provide sufficient sensing sites because of their flower-like morphology; at the same time, thanks to the versatile surface property of ND, the specific modification of the sensor can be easily implemented. Moreover, the high values in dielectric constant possessed by MNF and ND help to improve the intrinsic RI sensitivity, which are studied by both experiments and theories. The sensitivity improvement in biological detection was demonstrated by the IgG antigen-antibody binding process in a PBS solution, where the sensor showed good specificity as well. Besides, the practical application of a sensor for pseudorabies virus (PRV) in serum solution was also demonstrated.

## 2. Materials and Methods

### 2.1. Materials and Reagents

A combination of 3-mercaptopropionic acid (MPA), N-hydroxysuccinimide (NHS), 1-ethyl-3-(3-dimethylaminopropyl) carbodiimide (EDC), and ethanolamine were utilized to chemically modify the surface of SPR chip. Hexaammonium heptamolybdate tetrahydrate ((NH_4_)_6_Mo_7_O_24_·4H_2_O) and thiourea (CH_4_N_2_S) were used to synthesize MNF. The materials mentioned above were brought from the Shanghai Aladdin Company. Carboxylated NDs with an average size of 35 nm were purchased from the Taiwan FND Biotech Company. PBS was purchased from the Shanghai Sangon Biotech Company. Goat-anti-mouse IgG, mouse IgG, rabbit IgG, and bovine IgG were purchased from the Beijing Biosynthesis Biotechnology Company. PRV, PRV antibody, and pig serum samples were extracted from samples collected on the farm. PRV and PRV antibodies were dissolved in a mixture of pig serum and PBS (3:100 *v*/*v*). IgG reagents, PRV, PRV antibody, and serum sample were stored at −20 °C, and other reagents were stored at −4 °C.

### 2.2. Synthesis of MNF

MNF powders were synthesized by the one-pot hydrothermal method. Firstly, the homogeneous solution was prepared by mixing 1.24 g of hexaammonium heptamolybdate tetrahydrate [(NH_4_)_6_Mo_7_O_24_·4H_2_O] and 2.28 g of thiourea in 35 mL deionized water. The received homogeneous solution was then transferred into a 50-mL Teflon-lined stainless-steel autoclave. The temperature in the autoclave was increased to 200 °C for about 12 h, then cooled to room temperature. Finally, the acquired black product was rinsed repeatedly with deionized water and absolute ethanol and then dried at 60 °C for 12 h.

### 2.3. SPR Chips Preparation

SPR chips were fabricated on tailored silica slides at 28 × 20 × 1 mm (Jiuyi Optics, Fuzhou, China). The slides were first washed several times with deionized water and absolute ethanol. We have investigated the effect of gold film thickness [31] on the performance of SPR sensors, and according to the relevant results, the best performance of SPR sensors was obtained with a gold film thickness of 50 nm. So, a 5 nm thickness chromium layer and a 50 nm gold layer were successively coated on the slide surface by vacuum thermal evaporation, where the chromium layer was used to strengthen the adhesion of the gold layer on the slide. According to our previous related work [30], continued deposition of ND on a chip deposited once with MoS_2_, the MNF and ND dispersion solutions with the respective concentrations of 0.025 mg/mL and 0.05 mg/mL were prepared in ethanol/water (1:1 *v*/*v*) solvent with the aid of sufficient sonication for at least 30 min. Then a 200 μL MNF or ND dispersion solution was deposited onto the gold film and dried at room temperature for at least 12 h. Through repeating the above process of drop-casting and drying, multilayers of MNF or ND can be attached to the gold surface of the SPR chip.

### 2.4. Specific Modification on Chip Surface

**Figure 1:**
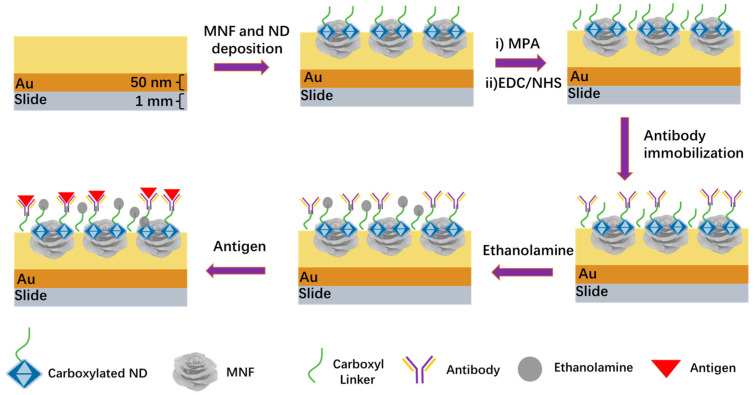
Schematic diagram of chemical modification and analyte detection on the chip surface.

Figure 1 shows the modification process and detection diagram. The chip coated with MNF and ND was first incubated in PBS solution for 30 min to soak the chip surface. Then, 10 mM MPA solution was used to replace PBS and incubated for about 60 min. The MPA was washed away by PBS, and then the EDC/NHS (1:1 *v*/*v*) mixture was added to the chip to activate the carboxyl groups on the gold surface. Afterward, 150 μg/mL antibody solution was incubated on the chip surface for 120 min and then washed with PBS. In the cases of IgG and PRV detection, the goat-anti-mouse IgG and PRV antibodies were employed, respectively. The chip was specifically functionalized as a result. Finally, 1 M of ethanolamine solution was added to occupy the remaining blank sites, suppressing nonspecific binding in the following detection process.

### 2.5. IgG Immunoassay

**Figure 2:**
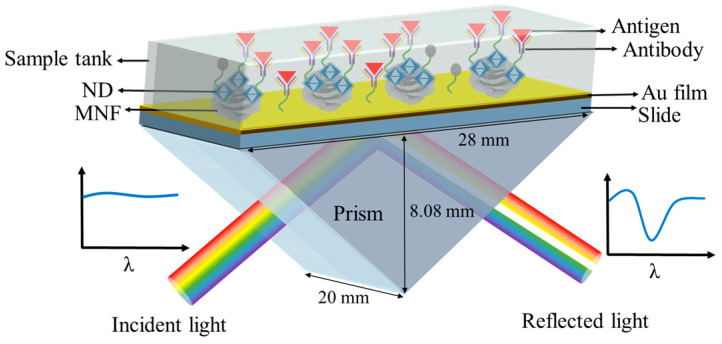
Schematic diagram for SPR immunoassay where the sensor chip was mounted on a prism.

The sensor chip immobilized with goat-anti-mouse IgG was exposed to mouse IgG solutions with different concentrations (5, 10, 20, 50, 100, 150, and 200 μg/mL) to measure immunoassay performance. Mouse IgG solutions were measured from low-to-high concentration successively, and each concentration solution was incubated on a chip surface for 30 min. Then chip surface was washed with PBS several times before proceeding to the next concentration. Each concentration was measured 3 times to evaluate the repeatability. The transmission spectrum of the sensor was real-time detected and recorded by a wavelength-interrogated SPR test system (see Section S1 in Supporting Information for details), where the SPR chip was mounted on a prism, as shown in Figure 2.

### 2.6. PRV Immunoassay

In addition, the SPR chip modified with PRV antibody was applied to PRV detection in a serum environment. PRV solutions with different concentrations (0, 0.5, 1.25, 2.5, 5, and 10 μg/mL), prepared in the solvent of pig serum and PBS mixture (3:100 *v*/*v*), were used to measure immunoassay performance. PRV solutions were detected from low to high concentration successively, and each concentration solution was incubated for 30 min. Before proceeding to the next concentration, the chip surface was flushed with PBS several times and then incubated in PBS until the measured signal stabilized.

## 3. Results and Discussions

### 3.1. Materials and Chips Characterization

**Figure 3:**
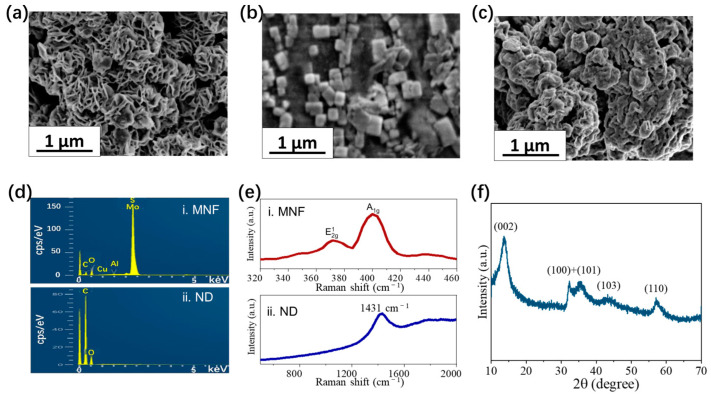
Characterization of materials. SEM images of (**a**) MNF, (**b**) ND, and (**c**) the chip surface deposited with the mixture of MNF and ND. (**d**) XPS and (**e**) Raman spectra of MNF and ND. (**f**) XRD spectrum of MNF.

MNF and ND are characterized by scanning electron microscope (SEM), X-ray photoelectron spectroscopy (XPS), Raman spectrometer, and X-ray diffraction (XRD). The SEM image of MNF (Figure 3a) shows the petal-like morphology exposing more faces and edge sites. The particle size of nanoflowers is about 0.5–1 µm. NDs in the SEM image (Figure 3b) show a polyhedron morphology, and the particle size ranges from 30 to 300 nm due to a certain degree of agglomeration. The SEM image on the chip surface deposited by the mixture of nanoflowers and NDs (Figure 3c) indicates that the gap between petals is occupied by NDs. The element composition of materials is demonstrated by XPS (Figure 3d). It also reveals that MNF contains a small number of impurity elements, such as Al and Cu, and ND contains oxygen elements due to the carboxyl functional groups on its surface. Besides, two Raman peaks at 378 cm^−1^ and 403 cm^−1^ can be observed for MNF (Figure 3e(i)), which is consistent with what has been reported in other articles [32], and one observed peak for NDs is located at 1431 cm^−1^ (Figure 3e(ii)). The measured diffraction peaks of MNF in the XRD spectrum (Figure 3f) are matched with the standard pattern of hexagonal MoS_2_ (JCPDS card No. 73-1508) [33]. The intensity ratio of diffraction peak (100) to (002) is 0.51, which means a large number of crystal faces (100) nanoflowers are exposed [34].

**Figure 4:**
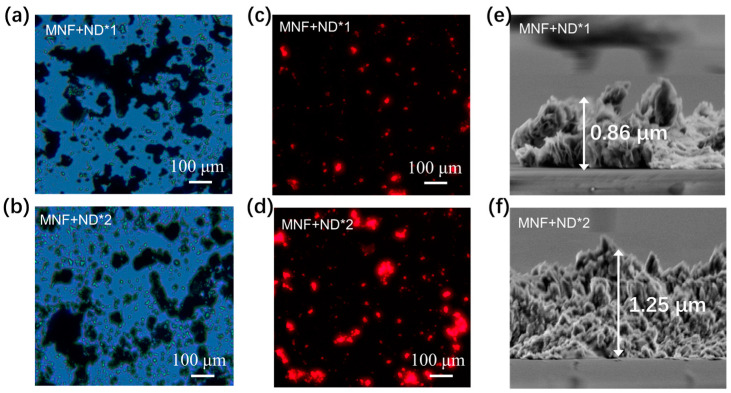
Characterization of sensor chips. (**a**,**b**) Optical microscope images, and (**c**,**d**) Corresponding fluorescent images on the chip’s surface deposited with MNF + ND*1 and MNF + ND*2, respectively. The NDs used in our experiments contain a nitrogen vacancy (NV) center, which emits red fluorescence under the excitation of green light [35]. This enables us to evaluate the amount of NDs modified on the chip surface based on the fluorescence intensity. (**e**,**f**) SEM images of chips interface from the side view.

The chip’s surface, deposited with MNF and ND, was characterized. Here, we used MNF + ND*n (*n* = 0,1,2…) to indicate that the chip surface was deposited with MNF for 1 time and ND for *n* times. Optical microscope images (Figure 4a,b) suggest the MNF and ND were attached to the chip surface. Moreover, the amount of ND particles increases with the modification times, verified by the increased fluorescence intensity emitted from NDs, as shown in Figure 4c,d. As a control, a chip surface modified with MNF only was characterized, and no NDs and fluorescence emission can be observed (Figure S2). SEM images on the cross-section of the chip surface (Figure 4e,f) show that the further deposition of ND on MNF will result in the thickness increase of the overlayer.

### 3.2. Bulk RI Sensitivity

**Figure 5:**
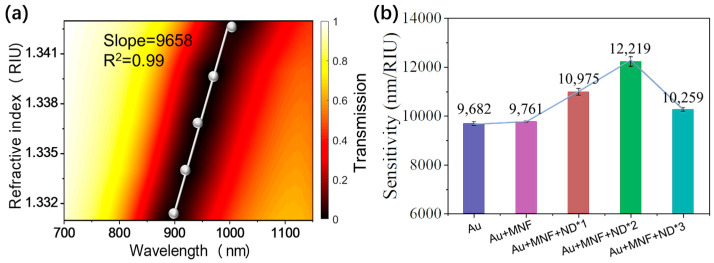
(**a**) Measured transmission spectra under varied surrounding RIs for Au chip. RI sensitivity is obtained by linearly fitting the resonant wavelength and RI. (**b**) Measured sensitivities for different SPR sensor chips.

Bulk sensitivity is a significant parameter to evaluate the performance of an SPR sensor. The bulk RI sensitivity of sensor chips deposited with MNF and ND at various times was characterized by varying the surrounding RI from 1.331 to 1.342 RIU (Figure 5). For each chip, the resonant wavelength shifts to a longer wavelength as the RI increases, but the wavelength shift amounts, namely RI sensitivities, are different, as shown in Figure 5 and Figure S3. The sensitivity gradually increases as MNF and ND are successively deposited on chips, reaching the highest when ND is deposited 2 times. However, the further deposition of ND to 3 times will cause a decrease in sensitivity. Compared with the initial sensitivity of 9682 nm/RIU for the bare Au chip, the highest sensitivity of 12,219 nm/RIU for the Au + MNF + ND*2 chip shows an enhancement of 26.2%. The sensitivity improvement can be attributed to the electric field, namely sensing field enhancement induced by the co-engineering of MNF and ND on the Au surface (see Section S4 in Supporting Information for details). On the other hand, the further deposition of ND results in the increase of thickness of the overlayer on the Au surface, thus reducing the overlap between the sensing field and analyte solution, which leads to the sensitivity degradation for the chip Au + MNF + ND*3 in our case.

### 3.3. IgG Immunoassay Performance

**Figure 6:**
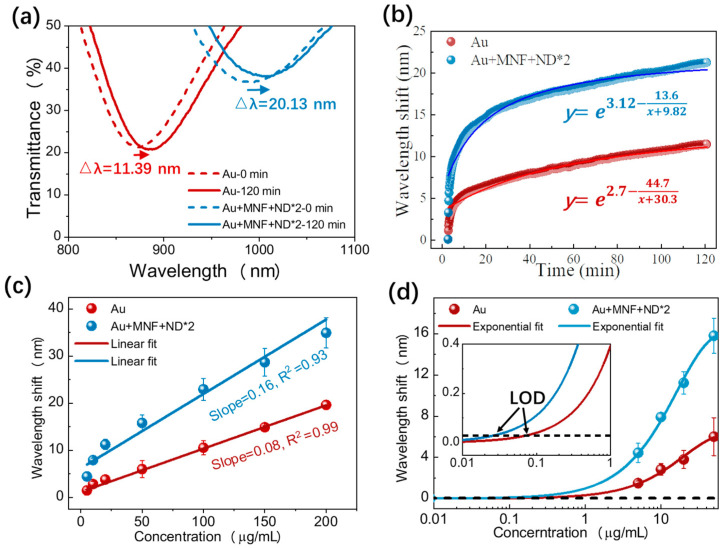
(**a**) Transmission spectra of the Au and Au + MNF + ND*2 chips incubated with 150 μg/mL goat-anti-mouse IgG solution at 0 and 120 min. (**b**) Real-time resonant wavelength shift during incubation. (**c**) Wavelength shifts of the two chips at varied mouse IgG concentration solutions. (**d**) Exponential fits at low concentration range.

Here, the sensor chips were designed for specific detection of mouse IgG. The chemically-modified Au chip and Au + MNF + ND*2 chip were first incubated with 150 μg/mL goat-anti-mouse IgG for 120 min. As the antibodies immobilized on the chip surface, the resonant wavelength shifted red (Figure 6a,b). Moreover, the shift amount for Au + MNF + ND*2 chip (20.13 nm) was significantly higher than that for the Au chip (11.39 nm). This is not only due to the higher bulk RI sensitivity of Au + MNF + NDs*2 but also because the deposited MNF and ND on the Au surface provide more antibody loading sites.

Then, the chips immobilized antibodies were incubated with mouse IgG solutions under varied concentrations to evaluate immunoassay performance. The dependence of resonant wavelength shift on concentration is presented in Figure 6c. As the concentration increased, the resonant wavelength shift increased due to more antigens linked on the chip surface. For a given antigen concentration, Au + MNF + ND*2 chip showed a larger redshift because of the improved RI sensitivity and antibody loading amount of MNF and ND. Linear fittings were carried out within the whole measurement range to evaluate the IgG detection sensitivity, and the sensitivity improved from 0.08 to 0.16 nm/(μg/mL) after MNF + ND*2 were deposited. Correspondingly, the limit of detection (LOD), defined as three times the standard deviation of the blank measurement, was lowered from 0.069 to 0.024 μg/mL [36].

**Figure 7:**
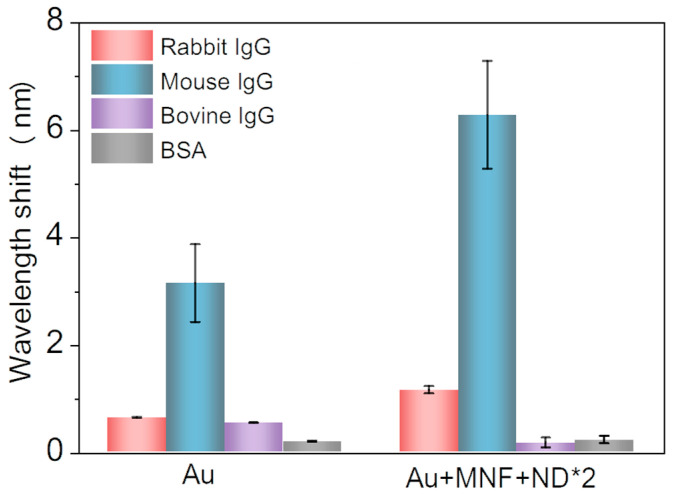
Measurement results of specificity experiments. Resonant wavelength shifts induced by different proteins under a fixed concentration for Au and Au + MNF + ND*2 chips.

To evaluate the specificity, the goat-anti-mouse IgG functionalized Au and Au + MNF + ND*2 chips were incubated with rabbit IgG, bovine IgG, mouse IgG, and BSA in PBS solution, respectively. Compared with the case of mouse IgG, the wavelength shifts induced by all the other analytes were much less for both the Au and Au + MNF + ND*2 chip (Figure 7), demonstrating a good specificity of the proposed immunoassay.

To have a more complete assessment of the proposed sensor, a comparison of the reported nanomaterials-modified SPR sensors in RI sensitivity and biological LOD is summarized in Table 1. Our proposed MNF and ND co-engineered sensor not only possesses much higher RI sensitivity but also shows a competitive LOD for IgG immunoassay. This is mainly attributed to the deposited MNF and ND modulating the plasmonic surface and enhancing the sensing field, and their special petal-like morphology exposing more faces and edge sites for antibodies to bind more antigens.

### 3.4. PRV Immunoassay Performance

**Figure 8:**
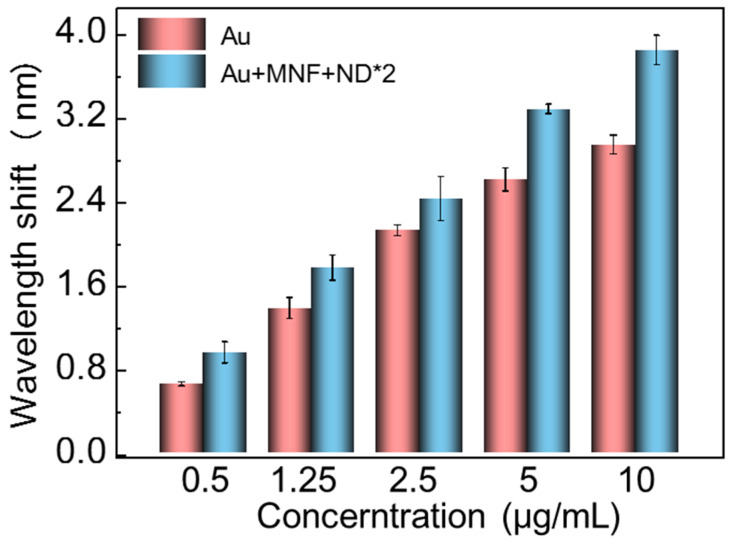
Resonant wavelength shifts of Au and Au + MNF + ND*2 chips at different PRV concentrations in serum solutions.

Porcine pseudorabies caused by PRV is an acute septic epidemic to which livestock and wildlife are susceptible, and developing a detection method for the diagnosis of wild-type PRV infection is important [44]. Here, our proposed SPR sensing scheme has also been demonstrated for sensitivity-enhanced PRV detection in a serum environment. PRV antibody was immobilized on the chip surface for immunoassay of PRV. The Au and Au + MNF + NDs*2 chips were incubated with 40 μg/mL PRV antibody for 60 min. The resonant wavelength shifts over time are shown in Figure S5. As similar to the case of goat-anti-mouse IgG, the PRV antibody immobilization-induced shift amount for Au + MNF + NDs*2 chip (11.49 nm) was larger than that for the Au chip (8.02 nm). Then, the two chips were incubated with different concentrations of PRV in serum solutions. The redshifts at different concentrations from 0.5 to 10 μg/mL were presented in Figure 8. Both chips perform an accumulated increase of resonant wavelength shift as the increase of concentration. Moreover, the Au + MNF + NDs*2 chip shows more redshift amount at each concentration. As a result, the LOD was lowered from 0.024 μg/mL (Au chip) to 0.018 μg/mL (Au + MNF + ND*2 chip), which was far lower than the proposed result by direct immunoassay of PRV [44].

## 4. Conclusions

In summary, an MNF and ND co-engineered SPR scheme was proposed and applied to the immunoassay of IgG and PRV. The scheme can be easily implemented by physically depositing an MNF and ND overlayer on the gold surface of an SPR chip, and the overlayer can be flexibly adjusted by controlling the deposition times to approach the optimal performance. The sensing performances of bulk RI, IgG, and PRV were investigated. It was found that the optimal deposition condition was MNF + ND*2, which improved the bulk RI sensitivity from 9658 to 12,219 nm/RIU. When applied to the IgG immunoassay, the Au + MNF + ND*2 chip showed a higher sensitivity of 0.16 nm/(μg/mL) than the 0.08 nm/(μg/mL) for Au chip, correspondingly lowering the LOD from 0.069 to 0.024 μg/mL. Characterization and simulation results showed that the improvement arose from the enhanced sensing field and increased antibody loading by the deposition of MNF and ND on the Au surface. The specificity of the sensor was also demonstrated through experimentation. Besides, the sensor was realistically applied to PRV immunoassay in a serum environment, and the LOD of 0.018 μg/mL for Au + MNF + ND*2 chip was achieved. This value was far lower than the proposed result by direct detection of PRV. The excellent performance of the proposed sensor is owing to the high RI and rich edge sites that provide more sites for the immobilization of a larger amount of antibodies. Because of its simple scheme and easy implementation, the proposed sensor provides an efficient and high-performance platform for biological detection.

## Figures and Tables

**Table 1:** Comparison with nanomaterial-modified SPR sensors for IgG immunoassay.

Nanomaterial	RI Sensitivity (nm/RIU)	Analyte	LOD (μg/mL)	Reference
Graphene oxide	3311	Human IgG	0.05	[37]
Reduced graphene oxide	-	Rabbit IgG	0.0625	[38]
Gold nanorod-iron oxide	-	Mouse IgG	0.15	[39]
MoSe_2_	2793	Rabbit IgG	0.33	[40]
Ag nanocubes	-	Mouse IgG	0.6	[41]
Half-antibody fragments	1959	Mouse IgG	0.1	[42]
Silver nanocubes	-	Human IgG	0.075	[43]
MNF + ND	12,219	Mouse IgG	0.024	This work

## Data Availability

We would happily provide the data upon request.

## Supplementary Materials

The following supporting information can be downloaded at: https://www.mdpi.com/article/10.3390/bios13050506/s1. Figure S1: Schematic diagram of the SPR test system; Figure S2: Characterization section; Figure S3: Bulk RI test results; Figure S4: Simulations; Figure S5: PRV measurement.
