# Supplementary material for: MoS2-Nanoflower and Nanodiamond Co-Engineered Surface Plasmon Resonance for Biosensing
Source: Biosensors (Basel). 2023 Apr 28;13(5):506. doi: 10.3390/bios13050506 (PMC10216570; doi:10.3390/bios13050506)
Supplement: Supplementary file 1 [file biosensors-13-00506-s001.zip › biosensors-2300140-supplementary.pdf]

biosensors

Supplementary Materials

MoS2-nanoflower and nanodiamond co-engineered surface plasmon resonance for biosensing

Yaofei Chen [1,2], Xin Xiong [1,2], Yu Chen [1,2], Lei Chen [1,2], Guishi Liu [1,2], Wei Xiao [3,*], Jifu Shi [4], Zhe Chen [1,2], Yunhan Luo [1,2,*]

1 Guangdong Provincial Key Laboratory of Optical Fiber Sensing and Communications, Jinan University, Guangzhou 510632, China
2 Department of Optoelectronic Engineering, Jinan University, Guangzhou 510632, China;
3 Department of Laboratory Medicine, Guangdong Second Provincial General Hospital, Guangzhou 510317, China;
4 Siyuan Laboratory, Department of Physics, Jinan University, Guangzhou 510632, China
* Corresponding author: xkevent@foxmail.com (W. Xiao), yunhanluo@163.com (Y.H. Luo)

1. SPR test system

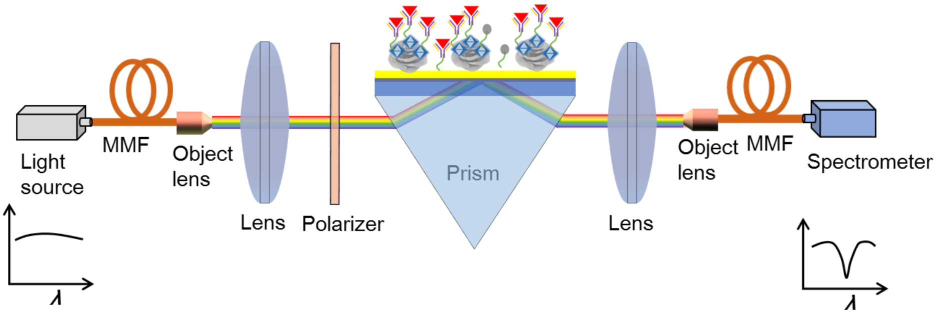

Figure S1. Schematic diagram of the SPR test system.

All the measurements in our experiments are carried out using a homemade wavelength-interrogation SPR test system (*Optics Express, 2018, 26(26): 34250-34258*). Figure S1 shows the schematic diagram of the proposed SPR sensor and the corresponding test system. The incident light, emitted from a tungsten-halogen lamp light source (AvaLight-HAL-(S)-Mini, China), propagates through a piece of multimode fiber (MMF) and is collimated by an objective lens and a convex lens. A polarizer is used to generate the transverse-magnetic wave. Then, the output light is recorded by a spectrometer (AvaSpec-ULS2048XL, China) with the aid of a convex lens and an object lens. The measured data are finally sent to a computer for further processing.

2. Characterization section

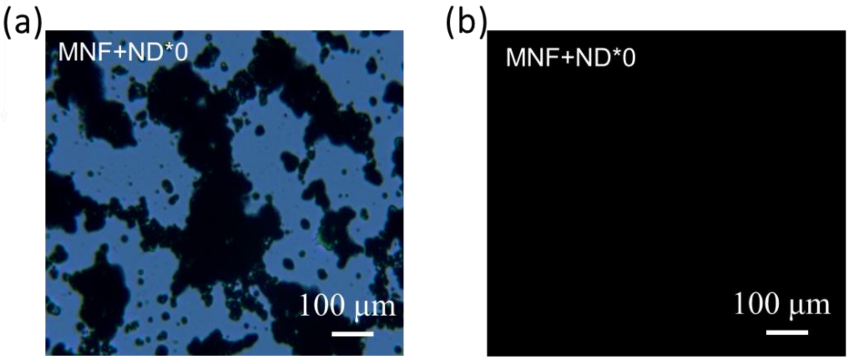

Figure S2. (a) Optical microscope image and (b) Fluorescence microscope image on the SPR chip deposited with MNF for one time.

3. Bulk RI test results

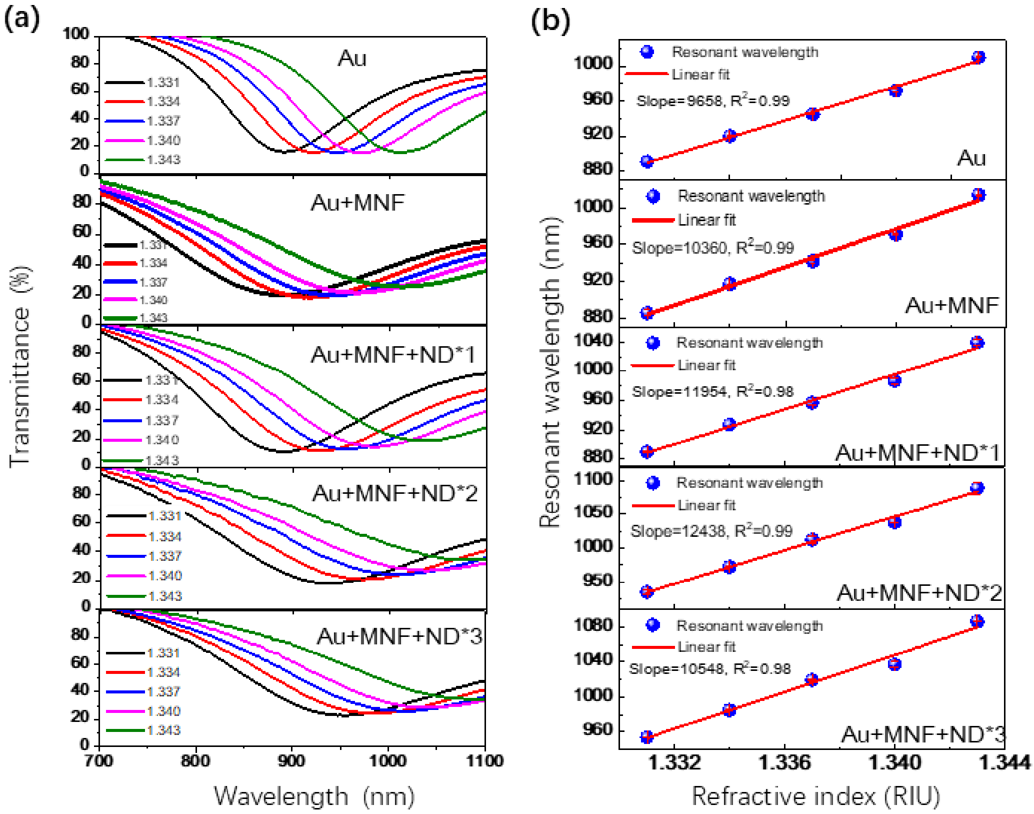

Figure S3. (a) Transmission spectra under varied surrounding RI environments for the SPR chips of Au, Au+MNF, Au+MNF+ND*1, Au+MNF+ND*2, and Au+MNF+ND*3, respectively. (b) Dependences of resonant wavelength on the surrounding RI and the corresponding linear fit results for the varied SPR chips.

4. Simulations

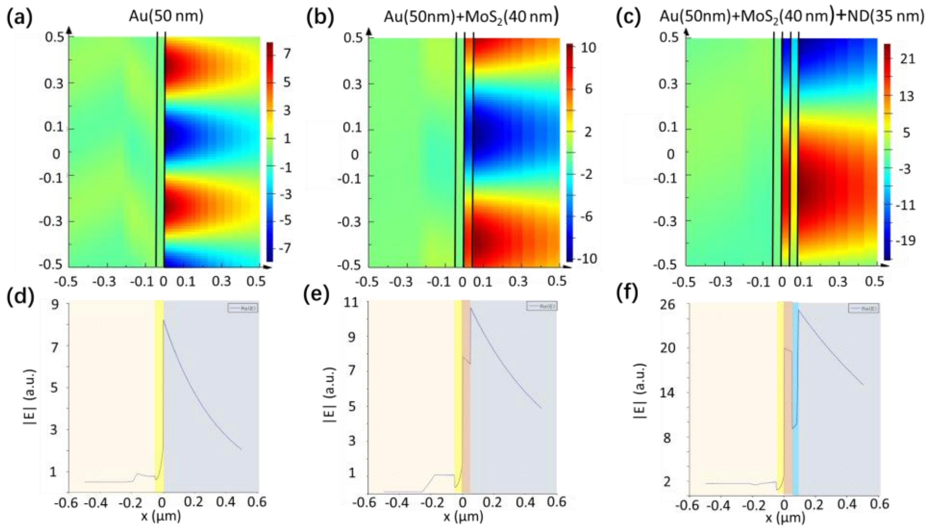

Figure S4. Simulated distributions of (a)-(c) The electrical field component in the x-direction in 2D and (d)-(f) The corresponding electrical field amplitude in 1D along the white dash lines in (a)-(c). (a)(d) Au chip, (b)(e) Au+MNF chip, (c)(f) Au+MNF+ND chip. The thicknesses of MNF and ND

layers (tens of nanometers) employed in simulations are much smaller than the measured values (hundreds of nanometers) shown in Figure 4e,f. This is reasonable since the measured thickness of an overlayer is the largest one presented in the SEM picture, and the deposited MNF and ND layers feature a porous and loose morphology.

The dielectric constant of the gold film is set to the Drude model [Journal of applied physics, 2007, 101(9): 093111.]. The distribution of MoS_2 on the gold film is not uniform, so the dielectric constant of MoS_2 is referred to in this work [Applied Physics Letters, 2014, 105(20): 201905.]. The electrical field distributions near the SPR chip surface were simulated based on the finite difference time domain method (Advanced Optical Materials, 2019, 7(13): 1900479). The simulation results are presented in Figure S4, where panels a–c show the 2D distribution of the electrical field component in the x direction for the Au, Au+MNF, and Au+MNF+ND chips, respectively, and panels d,f) present the distribution of the amplitude of electrical field in 1D. We can see that the electric field assembly distributes near the interface of the chip/analyte solution and penetrates the analyte solution for several hundred nanometers. It also indicates that the additional MNF and ND overlayer can significantly enhance the electrical field, and the electrical field amplitude is enhanced from 8.2 to 10.5 and 25.1 after the successive deposition of MoS_2 and ND. At the same time, the penetration depth of the electrical field into the analyte solution is also improved. All of these enhance the feeling strength of the sensing field to the surrounding solution, resulting in improved sensitivity.

5. PRV measurement

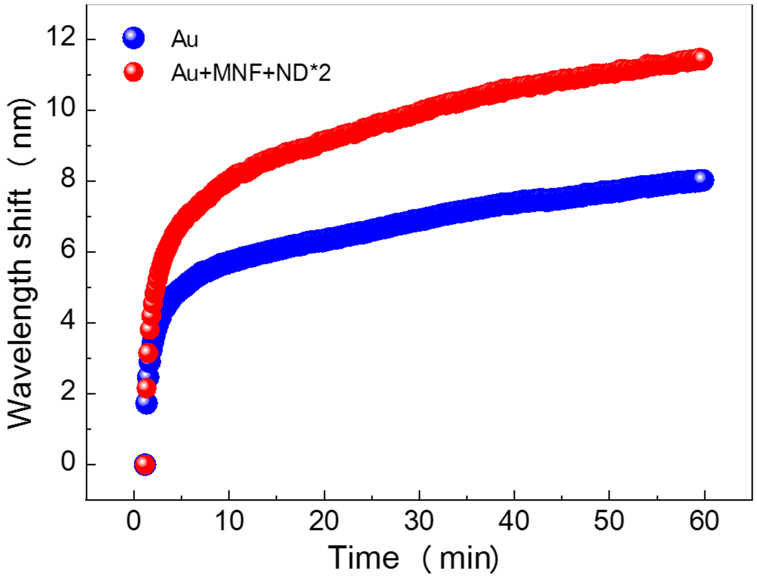

Figure S5. Real-time resonant wavelength shift during incubation of Au and Au+MNF+ND*2 SPR chips with PRV antibody solutions.
